# The Transcription Factor Nrf2 Mediates the Effects of *Antrodia camphorata* Extract on Neuropathological Changes in a Mouse Model of Parkinson’s Disease

**DOI:** 10.3390/ijms24119250

**Published:** 2023-05-25

**Authors:** Marika Lanza, Laura Cucinotta, Giovanna Casili, Alessia Filippone, Rossella Basilotta, Anna Paola Capra, Michela Campolo, Irene Paterniti, Salvatore Cuzzocrea, Emanuela Esposito

**Affiliations:** Department of Chemical, Biological, Pharmaceutical and Environmental Sciences, University of Messina, 7 Viale Ferdinando Stagno D’Alcontres, 31, 98166 Messina, Italy

**Keywords:** Parkinson’s disease, neuroinflammation, oxidative stress, MPTP

## Abstract

Parkinson’s disease (PD) is a disorder that is characterized by progressive and selective neuronal injury and cell death. Recent studies have provided accumulating evidence for a significant role of the immune system and neuroinflammation in PD pathogenesis. On this basis, many scientific articles have highlighted the anti-inflammatory and neuroprotective properties of *Antrodia camphorata* (AC), an edible fungus containing various bioactive compounds. This study aimed to evaluate the inhibitory effect of AC administration on neuroinflammation and oxidative stress in a murine model of MPTP-induced dopaminergic degeneration. AC (10, 30, 100 mg/kg) was administered daily by oral gavage starting 24 h after the first administration of MPTP, and mice were sacrificed 7 days after MPTP induction. In this study, treatment with AC significantly reduced the alteration of PD hallmarks, increasing tyrosine hydroxylase expression and reducing the number of alpha-synuclein-positive neurons. In addition, AC treatment restored the myelination process of neurons associated with PD and attenuated the neuroinflammatory state. Furthermore, our study demonstrated that AC was able to reduce the oxidative stress induced by MPTP injection. In conclusion, this study highlighted that AC could be a potential therapeutic agent for the treatment of neurodegenerative disorders such as PD.

## 1. Introduction

Parkinson’s disease (PD) represents the second-most common neurodegenerative disease, affecting every year about 5 million people worldwide [1]. PD, as a motor disorder, presents symptoms that include tremors, stiffness, bradykinesia, and difficulty walking; however, the pathology presents several clinical signs that also involve cognitive disorders, behavioral complications, dementia, and impaired emotional recognition in the advanced stages [2]. The symptomatology is the result of a complex etiopathogenesis that includes multifactorial factors [3]. Indeed, both genetic and environmental factors are involved, causing degeneration of the neurons in the substantia nigra pars compacta [4], triggering a severe reduction of dopamine content in the midbrain [5].

Current therapy for parkinsonian patients aims to attenuate clinical symptoms through the administration of levodopa, a dopamine precursor, cholinergic antagonists, and monoamine oxidase B (MAO-B) inhibitors [6,7]. However, although conventional therapeutic approaches have proven useful in providing symptom relief, they do not represent a resolutive cure for PD. Therefore, it would be useful to investigate the factors underlying neurodegenerative processes to discover new therapeutic targets for the management of PD patients. In this context, literature data elucidates the key role of inflammation and oxidative stress (OS) in the onset and progression of neurodegenerative diseases, particularly PD [8].

In fact, neuroinflammatory mediators, synergistically with reactive oxygen species (ROS) and reactive nitrogen species (RNS), worsen central nervous system (CNS) homeostasis, leading to the progressive degeneration of neurons [9].

Hence, targeted inhibition of OS and inflammatory processes may represent an attractive therapeutic approach to slow down or delay the progression of PD. In this background, many scientific articles have highlighted the anti-inflammatory [10] and antioxidant activity [11] of *Antrodia camphorata* (AC), as well as its neuroprotective effects in a model of ischemic stroke [12,13]; however, no one has investigated the effect on PD. This fungus, which only grows on the brown heartwood of Cinnamomum kanehirae Hayata in Taiwan at altitudes between 450 and 2000 m, was traditionally used by Taiwanese aborigines as a prescription for alcohol toxicity and exhaustion. Chinese folk medicine also used the mushroom for liver diseases, food and drug intoxication, abdominal pain, hypertension, and itchy skin. It was not until 1990 that AC, also referred to as Niuchangchih, was reported as a new species deserving of scientific investigation. In preclinical studies, AC has been shown to have multiple biological activities; however, the active substances that contribute to biological functions still remain unclear [14]. The identification of the active ingredients from AC is necessary for a better use of this medicinal mushroom; 78 compounds have been identified and structurally elucidated; predominant in fruiting bodies are generally terpenoids in a large number (39 compounds). Other active compounds present are ubiquinone derivatives, maleic and succinic acid derivatives, benzene derivatives, and finally lignans. Moreover, accumulating data have shown that AC is a potent direct free radical scavenger [15,16,17]. Moreover, several pieces of evidence have revealed its therapeutic effects on various pathologies, thus improving patients’ clinical outcomes; indeed, its biological properties result in powerful antioxidant, anti-inflammatory, and antitumor activities, as demonstrated by several pieces of scientific evidence [18,19]. In the present study, we investigated the properties of AC extract, composed of all the active components, such as a significant portion of terpenoids, ubiquinone derivatives, maleic and succinic acid derivatives, benzene derivatives, and lignans, as reported by Geethangili et al. [14] (see supplementary sheet).

Therefore, on these grounds, the aim of this study was to investigate the anti-inflammatory and antioxidant effects of AC extract on neuroinflammation and oxidative stress associated with PD through the MPTP (1-methyl-4-phenyl-1,2,3,6-tetrahydropyridine) in vivo model.

## 2. Results

### 2.1. AC Treatments Reduced Loss of TH Expression and Accumulation of α-Syn

**Figure 1:**
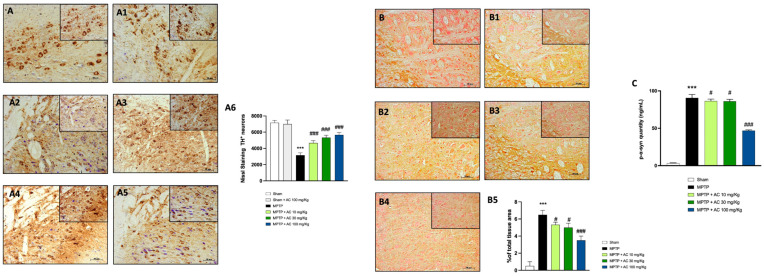
(**A**). Effect of AC treatment on TH expression in substantia nigra. MPTP group showed an extensive loss of TH neurons (**A2**), compared to the Sham group (**A**). AC treatment at the doses of 10, 30 and 100 mg/kg ((**A3**–**A5**), see score (**A6**)) significantly restored the number of TH neurons. Sham group treated with AC at dose of 100 mg/kg did not show any change (**A1**). One-way ANOVA test *** *p* < 0.001 vs. Sham; ### *p* < 0.001 vs. MPTP (**A6**). Effect of AC treatment on α-synuclein expression in substantia nigra (**B**,**C**). MPTP-intoxicated mice showed an increase in the number of α-syn aggregates in dopaminergic neurons (**B1**) compared to the Sham group (**B**). The neuroprotective role of AC at doses of 10, 30, and 100 mg/kg is revealed by the reduction in the number of α-syn aggregates (**B2**–**B4**). These results were confirmed by ELISA kit analysis of p-α-syn (**C**). One way ANOVA test *** *p* < 0.001 vs. Sham; # *p* < 0.05 vs. MPTP; ### *p* < 0.001 vs. MPTP (**B5**).

PD is characterized by a tyrosine hydroxylase deficiency, as TH catalyzes the formation of the DA precursor, L-DOPA [20]. To evaluate whether AC might protect against MPTP-induced loss of striatal DA neurons, midbrain sections were stained for tyrosine hydroxylase immunoreactivity. In the MPTP group, we observed a significant decrease in TH-positive neurons 7 days after MPTP injections compared to the sham group (Figure 1, respectively A2 and A; see score Figure 1(A6)). AC treatment at doses of 10, 30, and 100 mg/kg (respectively, Figure 1(A3–A5); see score Figure 1(A6)) restored TH expression levels. In addition, the sham group treated with AC at a dose of 100 mg/kg did not show any significant change compared to the sham group (respectively, Figure 1(A1,A); see score Figure 1(A6)) (See Supplementary Figure S1).

PD is characterized by an aggregation of citoplasmatic α-syn in the dopaminergic neurons of the substantia nigra [21]. To evaluate the neuroprotective effect of AC after MPTP injection, immunohistochemistry staining was performed on midbrain sections. An important increase in the number of α-syn aggregates in dopaminergic neurons was observed in the MPTP group compared to the sham group that showed basal levels of α-syn (Figure 1, respectively B1 and B; see score Figure 1(B5)). AC treatment at doses of 10 and 30 mg/kg (respectively, Figure 1(B2,B3), see score Figure 1(B5)), even more effectively at a dose of 100 mg/kg (Figure 1(B4), see score Figure 1(B5)), showed a significant decrease of α-syn-positive staining. To further assess the neuroprotective effect of AC against α-syn accumulation, we also evaluated the p-α-syn form as a peculiar protein implicated in the pathogenesis of PD. The MPTP group showed elevated p-α-syn levels compared to the Sham mice. AC, in a dose-dependent manner, decreased p-α-syn levels (Figure 1C); (see Supplementary Figure S1).

### 2.2. Effect of AC Treatments on Myelination Process

**Figure 2:**
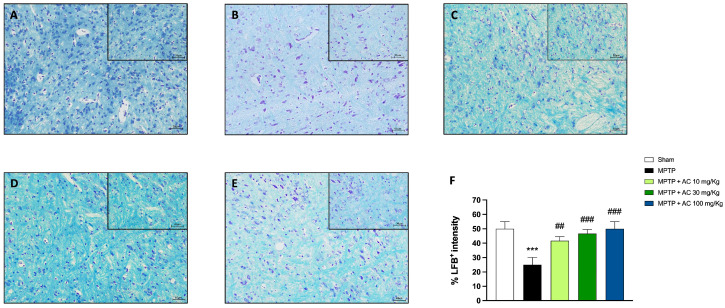
Effect of AC on the myelination process in substantia nigra. LFB staining showed a significant loss of myelin in MPTP group (**B**) compared to the Sham group (**A**). The treatment with AC at doses of 10, 30, and 100 mg/kg (**C**–**E**) significantly restored the myelin presence. One-way ANOVA test *** *p* < 0.001 vs. Sham; ## *p* < 0.01 vs. MPTP; ### *p* < 0.001 vs. MPTP (**F**).

LFB staining was performed to investigate the possible effect of AC on preserving neurons and myelin structure [22]. At 7 days after MPTP injection, a loss of myelin was observed in the MPTP group compared to the sham group (Figure 2, respectively B and A; see score in Figure 2F), in which myelin structure was clearly stained by LFB. The treatments with AC at doses of 10, 30, and 100 mg/kg (respectively, Figure 2C–E; see score in Figure 2F) significantly restored the myelin presence (see Supplementary Figure S2).

### 2.3. Effect of AC Treatments on GFAP and IBA-1 Expression

**Figure 3:**
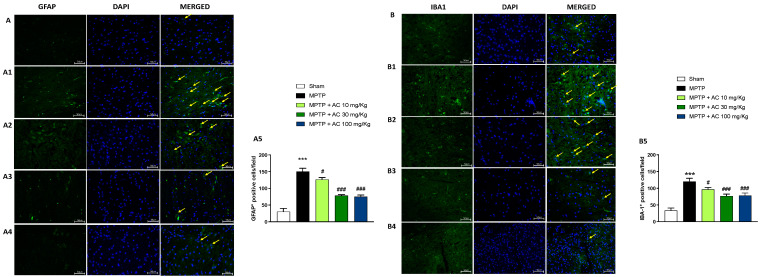
Effect of AC on GFAP, IBA-1 expression in substantia nigra. Midbrain section of MPTP-injected mice showed high expression of GFAP (**A1**) and IBA-1 (**B1**), compared to the Sham groups (**A**,**B**). GFAP and IBA-1 expression significantly decreased after AC treatments, especially at doses of 30 and 100 mg/kg (**A3**,**A4**,**B3**,**B4**). AC treatment at dose of 10 mg/kg (**A2,B2**). One way ANOVA test *** *p* < 0.001 vs. Sham; # *p* < 0.05 vs. MPTP; ### *p* < 0.001 vs. MPTP (**A5**,**B5**).

The nigrostriatal dopaminergic degeneration in neurons is accompanied by a substantial increase in astrocyte and microglia activation after MPTP injection [21], so we decided to evaluate the expression of the respective markers GFAP and IBA-1 by immunofluorescence staining. Our data showed an important increase in the number of GFAP and IBA-1 positive cells in MPTP groups compared to the sham groups (respectively, Figure 3(A1,A) and Figure 3(B1,B); see score Figure 3(A5,B5)). AC treatment at a dose of 10 mg/kg and even more at doses of 30 and 100 mg/kg significantly reduced GFAP and IBA-1 expression (respectively, Figure 3(A2–A4) and Figure 3(B2–B4); see score Figure 3(A5,B5)).

### 2.4. AC Modulated Pro-Inflammatory Cytokines Levels and NF-kB Pathway Induced by MPTP Intoxication

**Figure 4:**
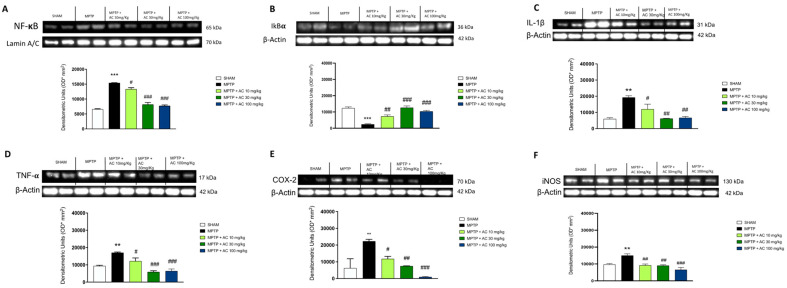
Effect of AC on NF-kB pathway and pro-inflammatory cytokine levels. Western blot analysis showed an important modulation of NF-kB (**A**), IkBα (**B**), and also of pro-inflammatory cytokines, such as IL-1β (**C**), TNF-α (**D**), COX-2 (**E**), and iNOS (**F**). One-way ANOVA test ** *p* < 0.01 vs. Sham; *** *p* < 0.001 vs. Sham; # *p* < 0.05 vs. MPTP; ## *p* < 0.01 vs. MPTP; ### *p* < 0.001 vs. MPTP.

In PD, neuroinflammation plays an important role in the neurodegenerative process and the consequent loss of dopaminergic neurons [23]. We decided to evaluate the expression of NF-κB, IκBα, COX-2, iNOS, TNF-α and IL-1β by Western blot analysis. We observed a basal expression of NF-κB in the sham group, an expression that was significantly increased in the MPTP group. Treatment with AC at doses of 10 mg/kg and even more effectively at doses of 30 and 100 mg/kg showed a significant decrease in NF-κB levels (Figure 4A). In the sham group, we observed basal levels of IκBα, on the contrary, IκBα expression was reduced in the MPTP group. AC at doses of 10, 30, and 100 mg/kg was able to prevent IκBα cytosolic degradation (Figure 4B).

Western blot analysis also showed a significant increase in IL-1β, TNF-α, COX-2, and iNOS levels in the MPTP group compared to the sham group, in which basal expression of them was found. However, AC treatment at doses of 10, 30, and 100 mg/kg significantly restored IL-1β, TNF-α, COX-2 and iNOS levels (Figure 4C–F).

### 2.5. Effect of AC Treatments on Antioxidant Response Activation

**Figure 5:**
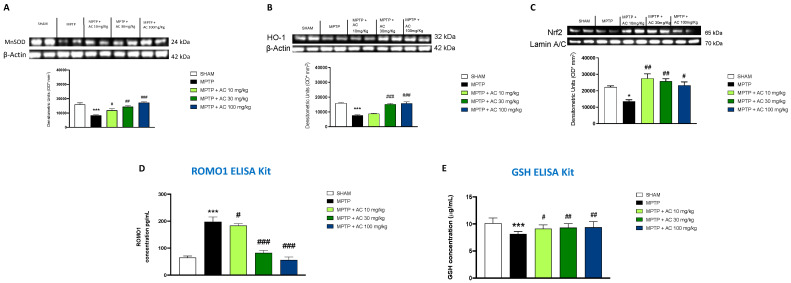
Antioxidant effect of AC. Western blot analysis showed an important modulation of MnSOD (**A**), HO-1 (**B**), and Nrf2 (**C**). ELISA kit showed an increase of ROMO1 levels (**D**) and a decrease of GSH (**E**) levels in MPTP group compared to the Sham group; AC restored their levels. One way ANOVA test * *p* < 0.05 vs. Sham; *** *p* < 0.001 vs. Sham; # *p* < 0.05 vs. MPTP; ## *p* < 0.01 vs. MPTP; ### *p* < 0.001 vs. MPTP.

In PD, oxidative stress plays a key role in determining neuronal cell damage [24], so we decided to evaluate the Nrf2 pathway and the relative antioxidant enzyme expression (MnSOD and HO-1) by Western blot analysis. The expression of MnSOD and HO-1 was significantly reduced after MPTP injection compared to the sham group. AC treatment at a dose of 10 mg/kg, even more effectively at doses of 30 and 100 mg/kg, showed a significant increase in MnSOD and HO-1 expression (Figure 5A,B).

The expression of Nrf2 levels showed a tendency to decrease after MPTP administration as compared with control mice. AC treatment at doses of 10, 30, and 100 mg/kg showed an increase in Nrf2 levels (Figure 5C).

Furthermore, we evaluated ROMO1 and GSH levels as additional markers of oxidative stress through a specific enzyme-linked assay (ELISA). ROMO1 levels were markedly higher in MPTP mice compared to the sham group, while the expression levels of ROMO1 were found to decrease after AC treatments at doses of 10, even more effectively at doses of 30 and 100 mg/kg. On the contrary, GSH levels were markedly lower in MPTP mice compared to the sham group, while the expression levels of GSH were found to increase after AC treatments at doses of 10, 30, and 100 mg/kg (Figure 5D,E). The results confirmed the ability of AC to reduce oxidative stress.

## 3. Discussion

PD is the second most common chronic neurodegenerative disorder, characterized by both motor and nonmotor symptoms, resulting from a pathophysiologic loss or degeneration of DA neurons in the midbrain substantia nigra pars compacta and the development of neuronal Lewy bodies [25]. Recent years have witnessed an increase in therapies for the treatment of PD based on the restoration of dopaminergic tone in the striatum; however, these therapies have not been able to modify the disease course or treat the non-dopamine-dependent features of PD, such as cognitive impairment and other non-motor features of the disorder, which often have the greatest impact on quality of life [26]. As understanding of PD pathogenesis grows, novel therapeutic avenues are emerging, and modification of the disease course by neuroprotective therapy is an important unmet clinical need. To this end, understanding the cellular mechanisms involved in PD is the subject of intensive research [27]. Although the pathogenesis of neuronal degeneration in PD is still to be fully understood, it is known that the increase in reactive oxygen species triggers a cascade of events leading to cell death; moreover, during a neuroinflammatory response, activated microglia produce nitric oxide and superoxide [28,29,30].

Several studies highlighted the capacity of AC to take part in several processes, such as inflammation; Hseu et al., in their study, demonstrated that AC exerts an anti-inflammatory effect by down-regulating iNOS and COX-2 expression through suppression of NF-κB activation in the pathophysiology of inflammatory diseases [10]. Moreover, the anti-oxidative role of AC is well known [15]. On these considerations, this study aimed to evaluate the protective role of AC in preventing dopaminergic cell death and glial activation in the MPTP model of PD in mice and investigate the molecular mechanisms by which AC protects mouse nigrostriatal neurons from MPTP-induced neurotoxicity and neuroinflammation.

A great deal of importance in the pathogenesis of PD is due to TH, a rate-limiting enzyme and one of the most important factors in catecholamine biosynthesis, including dopamine. Studies on experimental models of PD demonstrate that the reduction in DA metabolism-related markers such as TH is far greater than the loss of neuronal cell bodies [31]. In this study, treatment with AC significantly protected against MPTP-induced loss of TH+ neurons in the substantia nigra.

Furthermore, MPTP-induced neuronal degeneration is associated with the redistribution of α-syn from its normal synaptic location to aggregates in degenerating neuronal cell bodies, the early stages of Lewy body formation. α-Syn, a presynaptic neuronal protein, contributes to PD pathogenesis in a number of ways [32]. We highlighted that AC treatment notably reduced α-synuclein-positive neuron number. In addition, considering the phosphorylation of α-syn critical in PD pathogenesis, we evaluated its expression after AC treatment. Our data demonstrated a significant decrease of p-α-syn in AC-treated mice, validating once again the beneficial effects of this compound.

Recent studies have demonstrated the involvement of neuroinflammation in the pathophysiology of PD, revealing the involvement of chronic inflammation and microglia activation in the degeneration of dopaminergic neurons in PD patients [33]. Our results confirm an important increase in GFAP and IBA-1 expression following MPTP-induced nigrostriatal degeneration; on the contrary, AC administration reduced reactive astrocytes and microglia, restoring GFAP and IBA-1 levels. Han et al. suggest in their study that chronic activation of microglia and astrocytes may contribute to nerve fiber degeneration and loss of the myelin sheath [34]. Myelin alterations may represent a key pathological feature in neurodegenerative diseases, such as PD [35]. In this study, we demonstrated that AC treatment significantly protected the brain from demyelination by restoring myelin content.

The neuroinflammatory response is primarily mediated by the transcription factor NF-κB, which is activated by pro-inflammatory signals and controls the gene expression of most of the inflammatory mediators produced by microglial cells. In this study, we showed the capacity of treatment with AC to inhibit the nuclear translocation of NF-κB while promoting the transcription of IκB-α, thus representing a functional system for the regulation of neuroinflammation. During inflammation, the pro-inflammatory NF-κB induces the up-regulation of COX-2, iNOS, TNF-α, and Il-1β [22]. We demonstrated that AC treatment significantly reduced the expression of all inflammatory enzymes COX-2, Inos, TNF-α, and IL-1β. Recent findings have highlighted that NF-κB interferes with Nrf-2 in PD [24,36]. Nrf-2 represents a key regulator of endogenous inducible defense systems that, in response to oxidative stress, translocate to the nucleus and bind to specific DNA sites termed antioxidant response elements [37]. Interestingly, the regulation of Nrf-2 signaling has been shown to be a promising strategy to modulate the progression of the neurodegeneration associated with PD [38]. The treatment with AC upregulated the Nrf-2 transcriptional system, playing a major role in neuronal cell and tissue defense against oxidative stress. Nrf-2 induces phase II detoxifying and antioxidant enzymes, such as Mn-SOD and HO-1, which work together to fight oxidative stress and inflammation [39]. In this study, we showed that AC treatment, via the Nrf-2 pathway, reduces Mn-SOD expression and upregulates HO-1 expression, conferring resistance against neurodegenerative insults.

Neurons are especially vulnerable to oxidative stress, and several neurodegenerative diseases have been shown to have increased levels of oxidative stress [40]. Our data demonstrated the ability of AC to restore ROMO1, a marker of oxidative stress. Although, in most cases, the origin of the disease is unknown, PD has been associated with impairment in many of the neuroprotective mechanisms associated with the antioxidant response. Particularly, neuronal cells can activate antioxidant defense mechanisms involving GSH regulation. In human brains, the levels of GSH peroxidase correlate with the survival of dopaminergic neurons in PD, and reduced glutathione levels have been found in the brains of patients with PD [41]. We demonstrated that treatment with AC normalized GSH levels.

## 4. Materials and Methods

### 4.1. Materials

AC extract was purchased by Biogenerica (Catania, Italy); detailed datasheets are available from the manufacturer’s website. All other chemicals were of the highest commercial grade available. All stock solutions were prepared in non-pyrogenic saline (0.9% NaCl; Baxter, Rome, Italy).

### 4.2. Animals

Adult male CD1 mice (25–30 g, 10–12 weeks old; Envigo, Udine, Italy) were used for all studies. Mice were housed in cages (five per cage) and maintained at 21 ± 1 °C with a 12 h light and 12 h dark cycle. Standard laboratory diets and tap water were available ad libitum. Animal care was in compliance with Italian regulations on the protection of animals used for experimental and other scientific purposes (Ministerial Decree 116,192), as well as with the council regulation (EEC) (Official Journal of the European Union L 358/1 12/18/1986).

### 4.3. MPTP-Induced Nigrostriatal Degeneration

Animals received four intraperitoneal injections of MPTP (20 mg/kg; Sigma–Aldrich, St. Louis, MO, USA) in saline at 2 h intervals in one day, and the total dose for each mouse was 80 mg/kg. After 24 h after the first MPTP injection, animals received oral administration (OS) of AC extract at doses of 10, 30, and 100 mg/kg; AC was given once daily until 7 days after the MPTP injection. Mice were sacrificed 7 days after MPTP injection through an overdose of anesthetic, and their brains were surgically collected. Then, for histological assessments and molecular biology analyses, the midbrain area (substantia nigra) was evaluated. The dose of MPTP (80 mg/kg) used was based on previous in vivo studies [42].

### 4.4. Experimental Design

Mice were randomly distributed into the following groups:

- Group 1: Sham + vehicle: Vehicle solution (saline) was administered i.p. during the 1st day, similar to the MPTP protocol. (N = 8);
- Group 2: Sham + AC 10 mg/kg: same as the Sham + Veh group, but AC was administered orally starting 24 h after vehicle solution injection for 7 consecutive days (N = 8);
- Group 3: Sham + AC 30 mg/kg: same as the Sham + Veh group, in addition AC was administered orally starting 24 h after vehicle solution injection for 7 consecutive days (N = 8);
- Group 4: Sham + AC 100 mg/kg: same as the Sham + Veh group; in addition, AC was administered orally starting 24 h after vehicle solution injection for 7 consecutive days (N = 8);
- Group 5: MPTP + vehicle: MPTP solution was administered as described for administration of saline. (N = 8);
- Group 6: MPTP + AC 10 mg/kg; same as the MPTP + Veh group, but AC was administered orally starting 24 h after the first MPTP administration and continuing through 7 additional days after the last injection of MPTP (N = 8);
- Group 7: MPTP + AC 30 mg/kg; same as the MPTP + Veh group, but AC was administered orally starting 24 h after the first MPTP administration and continuing through 7 additional days after the last injection of MPTP (N = 8);
- Group 8: MPTP + AC 100 mg/kg; same as the MPTP + Veh group, but AC was administered orally starting 24 h after the first MPTP administration and continuing through 7 additional days after the last injection of MPTP (N = 8).

AC extract is composed of all the active components, such as a significant portion of terpenoids, ubiquinone derivatives, maleic and succinic acid derivatives, benzene derivatives, and lignans. The dose and route of administration of AC were based on previous in vivo studies [43] and on a dose-response pilot experiment, considering the mice’s body surface area-based dosing. Results for the AC-treated Sham groups were not shown because neither toxicity nor improvement were seen compared to the Sham + vehicle group.

### 4.5. Immunohistochemical Analysis

The immunohistochemical localization was executed as previously described [44]. In brief, all slides were incubated overnight (O/N) using the following primary antibodies: anti-tyrosine hydroxylase (TH) (1:100; Santa Cruz Biotechnology, Dallas, TX, USA; sc-25269), and anti-α-synuclein (α-syn) (1:100; Santa Cruz Biotechnology, Dallas, TX, USA; sc-7011-R). After washing with PBS, sections were incubated with a secondary antibody for 1 h at room temperature. The reaction was revealed by a chromogenic substrate (DAB) and counterstaining with nuclear fast red. The images were acquired using an optical AxioVision microscope (Zeiss, Germany). For graphic display of densitometric analyses, the % of positive staining (brown staining) was measured by computer-assisted color image analysis (Leica QWin V3, Cambridge, UK). For immunohistochemistry, the images were shown at 10× (100 μm of the bar scale; see supplementary file), 20× (50 μm of the bar scale), and 40× (20 μm of the bar scale).

Unbiased counting of TH+ dopaminergic neurons in the substantia nigra par compacta (SNpc) was performed by incubating any section with polyclonal primary antibody mouse anti-TH O/N and processing with the ABC method (Vector Laboratories, Burlingame, CA, USA). Brain sections were counterstained with cresyl violet, a Nissl stain. To count the number of TH+ cells, StereoInvestigator software (version 10) was used (Microbrightfield, Williston, VT, USA). The area of interest for counting TH-immunoreactive cells was performed within a 50 × 50 × 5 µm frame on the same side of the brain, with an upper and lower control zone of 1 µm; for Nissl cell counting, the same sections were examined.

### 4.6. Luxol Fast Blue (LFB) Staining

In order to assess the degree of myelination/demyelination, staining with the LFB stain kit (#ab150675, Abcam, Cambridge, UK) was performed in the deparaffinized sections following the manufacturer’s instructions [45]. In brief, midbrain sections were incubated in LFB solution at 56 °C O/N. Subsequently, after washing in 95% alcohol, the sections were incubated in a lithium carbonate solution and 70% ethyl alcohol and finally counterstained in the cresyl violet solution. Sections were assembled with Eukitt (Bio-Optica, Milan, Italy) and observed by light microscopy (AxioVision, Zeiss, Milan, Italy). The slides were analyzed by a pathologist blinded to the treatment groups. LFB positive counts were performed on each slide using an Axiovision Zeiss microscope (Milan, Italy). Images were shown by using an objective lens at 10 × (100 μm of the bar scale, see supplementary file), 20 × (50 μm of the scale bar), and 40 × (20 μm of the scale bar).

### 4.7. Immunofluorescence Staining

Brain sections were processed for immunofluorescence staining as previously described [46] and reported below. In brief, all sections were incubated overnight (O/N) in a humidified chamber at 37 °C using the following primary antibodies: anti-GFAP antibody (1:100; sc-33673; Santa Cruz Biotechnology), anti-IBA1 antibody (1:100; sc-32725; Santa Cruz Biotechnology). Sections were washed with PBS solution and incubated with IgG (H + L) highly cross-adsorbed goat anti-mouse secondary antibody, Alexa Fluor™ (1:1000 in PBS *v*/*v*, Molecular Probes, Altrincham, UK) for 1 h at 37 °C. After washing in PBS, nuclear staining with 4′,6′-diamidino-2-phenylindole (DAPI; Hoechst, Frankfurt, Germany) (2 µg/mL) in PBS was added. Slides were observed and photographed at 40× magnifications using a Leica DM2000 microscope (Leica QWin V3, Cambridge, UK).

### 4.8. Western Blot Analysis

Western blot analysis was performed on brain tissues harvested 7 days after MPTP injection. Cytosolic and nuclear extracts were prepared as described previously [47]. The expression of cyclooxygenase 2 (COX-2), inducible nitric oxide synthase (iNOS), tumor necrosis factor-α (TNF-α), interleukin-1β (IL-1β), manganese superoxide dismutase (Mn-SOD), heme-oxygenase (HO-1) and nuclear factor of kappa light polypeptide gene enhancer in B-cells inhibitor-α (IκB-α) was quantified in the cytosolic fraction. Nuclear factor kappa-light-chain enhancer of activated B cells (NF-κB) and nuclear factor erythroid 2-related factor 2 (Nrf-2) expressions were quantified in the nuclear fraction. The membranes were incubated at 4 °C overnight with specific primary antibodies: anti-COX2 (1:500; Santa Cruz Biotechnology, SC-376861), anti-iNOS (1:500; Santa Cruz Biotechnology, SC654), anti-TNF-α (1:500; Santa Cruz Biotechnology. SC-52746), anti-IL-1β (1:500; Santa Cruz Biotechnology. SC-32294), anti-MnSOD (1:500; Santa Cruz Biotechnology. SC-06984), anti-HO-1 (1:500; Santa Cruz Biotechnology. SC-136960), anti-IκB-α (1:500; Santa Cruz Biotechnology. SC-1643), anti-NF-κB (1:500; Santa Cruz Biotechnology. SC-8008) and anti-Nrf2 (1:500; Santa Cruz Biotechnology, SC-365949) in 1 × PBS, 5% *w/v* non-fat dried milk, 0.1% Tween 20 at 4 °C overnight. To ascertain that blots were loaded with equal amounts of proteins, they were also incubated in the presence of the antibody against β-actin protein (cytosolic fraction 1:500; Santa Cruz Biotechnology) or LAMIN A/C (nuclear fraction 1:500; Santa Cruz Biotechnology). Signals were detected with an enhanced chemiluminescence (ECL) detection system reagent according to the manufacturer’s instructions (Thermo Fisher Scientific, Waltham, MA, USA). The relative expression of the protein bands was quantified using a standardization to β-actin and lamin A/C levels. Images of blot signals (8 bit/600 dpi resolution) were imported into analysis software (Image Quant TL, v2003).

### 4.9. Mouse Romo1, GSH and p-α-syn ELISA Kit

ELISA kit assays for reactive oxygen species modulator 1 (ROMO1) and glutathione (GSH) were performed on brain tissue extracts for each experimental group as previously described [48]. ROMO1 and GSH were measured according to the manufacturer’s instructions (respectively, Aviva Systems Biology, San Diego, CA, USA and Cloud-Clone Corp, Katy, TX, USA), and their expression was evaluated by reading the OD absorbance at 450 nm in a microplate reader.

In addition, phospho-α-syn was evaluated on brain tissue extracts as previously described [21] by an ELISA kit, according to the manufacturer’s instructions (MyBioSource, Kuiper, The Netherlands), through a colorimetric microplate reader.

### 4.10. Statistical Analysis

All values are expressed as the mean ± standard error of the mean (SEM) of *n* observations. Each analysis was performed three times, with three samples replicated for each one. The results were analyzed with GraphPad 9 software by one-way analysis of variance (ANOVA), followed by a Bonferroni post hoc test for multiple comparisons. A *p*-value of less than 0.05 was considered significant.

## 5. Conclusions

In conclusion, these results indicate that AC, by modulating Nrf-2-mediated oxidative stress and Nf-κB inflammatory pathways, could represent a potential neuroprotective approach in the pathophysiological process of PD. These data suggested that AC administration could enhance the physiological antioxidant and anti-inflammatory response, which could be extended to other neurodegenerative diseases.

In light of this, future studies using in vivo knockout models will be able to establish in detail the activity of AC on the NF-κB/Nrf-2 pathway and in particular on Nrf-2 in the context of PD, thus evaluating its possible use as a therapeutic agent.

## Data Availability

The data that support the findings of this study are available from the corresponding author upon reasonable request.

## Supplementary Materials

The following supporting information can be downloaded at: https://www.mdpi.com/article/10.3390/ijms24119250/s1.

## Abbreviations

PD: Parkinson’s disease; TH: tyrosine hydroxylase; α-syn: α-Synuclein; CNS: central nervous system; AC: *Antrodia camphorata*; ROS: reactive oxygen species; MPTP: 1-methyl-4-phenyl-1,2,3,6-tetrahydropyridine; ROMO1: reactive oxygen species modulator; GSH: glutathione; COX-2: cyclooxygenase 2; iNOS: inducible nitric oxide synthase; TNF-α: tumor necrosis factor-α; IL-1β: interleukin-1β; Mn-SOD: manganese superoxide dismutase; HO-1: heme-oxygenase; IκB-α: nuclear factor of kappa light polypeptide gene enhancer in B-cells inhibitor-α; NF-κB: nuclear factor kappa-light-chain-enhancer of activated B cells; Nrf-2: nuclear factor erythroid 2-related factor 2.
