# Supplementary material for: The Transcription Factor Nrf2 Mediates the Effects of Antrodia camphorata Extract on Neuropathological Changes in a Mouse Model of Parkinson’s Disease
Source: Int J Mol Sci. 2023 May 25;24(11):9250. doi: 10.3390/ijms24119250 (PMC10252478; doi:10.3390/ijms24119250)
Supplement: Supplementary file 1 [file ijms-24-09250-s001.zip › ijms-2384411-supplementary.pdf]

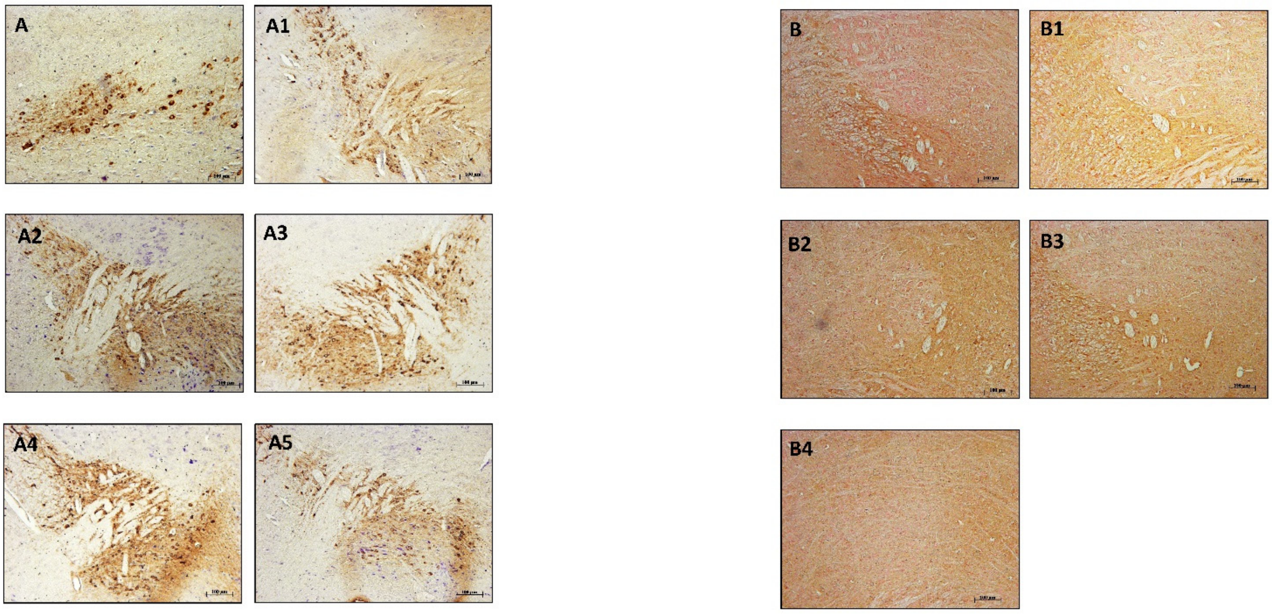

Supplementary Figure S1. Effect of AC treatment on TH (**A-A5**) and α-synuclein (**B-B4**) expression in substantia nigra. Images were shown by using an objective lens at 10 x (100 μm) of the bar scale. Sham group (**A-B**). Sham treated with AC 100 mg/kg (**A1**). MPTP group (**A2- B1**). AC treatments at the doses of 10, 30 and 100 mg/kg (**A3–A5** and **B2-B4**).

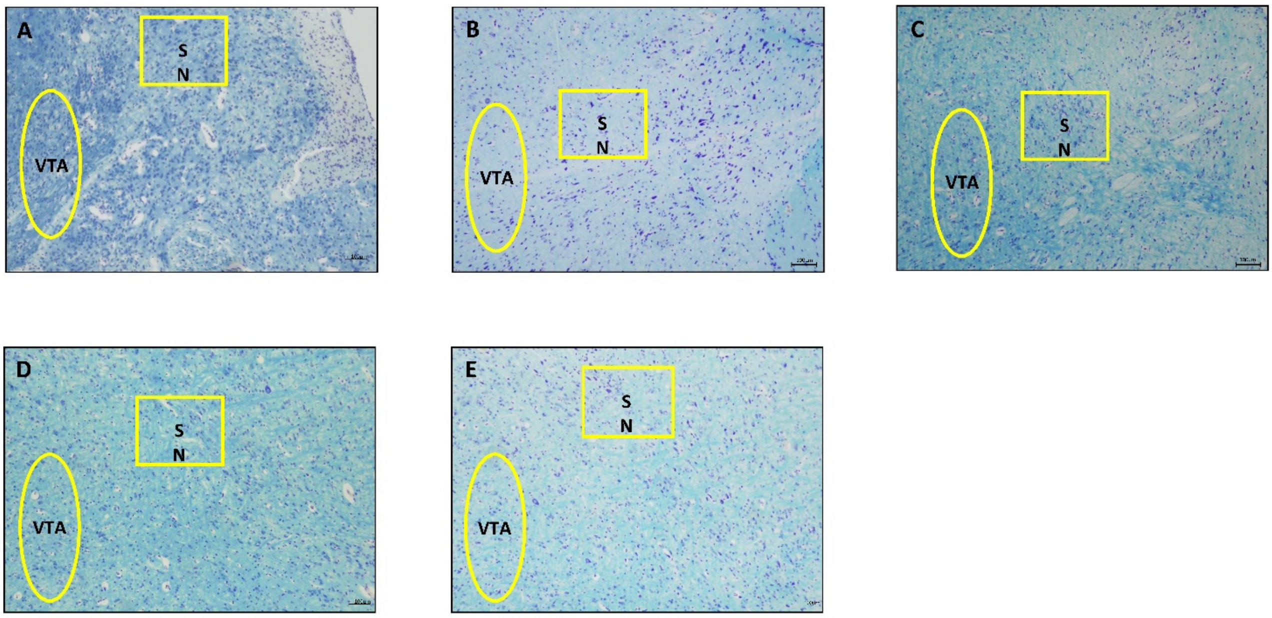

Supplementary Figure S2. Effect of AC on the myelination process in substantia nigra and VTA. Images were shown by using an objective lens at 10 x (100 μm) of the bar scale. Sham group (**A**) and MPTP group (**B**). AC treatment at the doses of 10, 30 and 100 mg/kg (**C-D-E**).
